# Exposure to 2.45 GHz Radiation Triggers Changes in HSP-70, Glucocorticoid Receptors and GFAP Biomarkers in Rat Brain

**DOI:** 10.3390/ijms22105103

**Published:** 2021-05-12

**Authors:** Haifa Othman, Alberto López-Furelos, José Manuel Leiro-Vidal, Mohamed Ammari, Mohsen Sakly, Hafedh Abdelmelek, Aarón Ángel Salas-Sánchez, Francisco Ares-Pena, Elena López-Martín

**Affiliations:** 1 Laboratory of Integrative Physiology, Faculty of Sciences of Bizerte, University of Carthage, 7021 Jarzouna, Tunisia; haifa.othman@fsb.rnu.tn (H.O.); mohamed.ammari@fsb.rnu.tn (M.A.); mohsensakly@gmail.com (M.S.); hafedh.abdelmelek@fsb.rnu.tn (H.A.); 2 Department of Morphological Sciences, Faculty of Medicine, University of Santiago de Compostela, E-15782 Santiago de Compostela, Spain; alberto.lopez.furelos@gmail.com; 3 Department of Microbiology and Parasitology, Research Institute on Chemical and Biological Analysis, University of Santiago de Compostela, E-15782 Santiago de Compostela, Spain; josemanuel.leiro@usc.es; 4 Higher Institute of Applied Biological Sciences of Tunis, University of Tunis El Manar, 9, Rue Zouhair Essafi, 1006 Tunis, Tunisia; 5 CRETUS Centre, Department of Applied Physics, Faculty of Physics, University of Santiago de Compostela, E-15782 Santiago de Compostela, Spain; aaronangel.salas@usc.es (A.Á.S.-S.); francisco.ares@usc.es (F.A.-P.); 6 ELEDIA Research Center, DISI, University of Trento, 38123 Trento-Alto Adige, Italy; 7 CRETUS Centre, Department of Morphological Sciences, Faculty of Medicine, University of Santiago de Compostela, E-15782 Santiago de Compostela, Spain

**Keywords:** 2.45 GHz radiation, GFAP, glucocorticoid receptor, HSP-70, nonionizing radiation

## Abstract

Brain tissue may be especially sensitive to electromagnetic phenomena provoking signs of neural stress in cerebral activity. Fifty-four adult female Sprague-Dawley rats underwent ELISA and immunohistochemistry testing of four relevant anatomical areas of the cerebrum to measure biomarkers indicating induction of heat shock protein 70 (HSP-70), glucocorticoid receptors (GCR) or glial fibrillary acidic protein (GFAP) after single or repeated exposure to 2.45 GHz radiation in the experimental set-up. Neither radiation regime caused tissue heating, so thermal effects can be ruled out. A progressive decrease in GCR and HSP-70 was observed after acute or repeated irradiation in the somatosensory cortex, hypothalamus and hippocampus. In the limbic cortex; however, values for both biomarkers were significantly higher after repeated exposure to irradiation when compared to control animals. GFAP values in brain tissue after irradiation were not significantly different or were even lower than those of nonirradiated animals in all brain regions studied. Our results suggest that repeated exposure to 2.45 GHz elicited GCR/HSP-70 dysregulation in the brain, triggering a state of stress that could decrease tissue anti-inflammatory action without favoring glial proliferation and make the nervous system more vulnerable.

## 1. Introduction

The use of radiofrequencies is increasing exponentially in different fields such as medicine [1], wireless communication devices and networks, and even space missions [2].

Extensive, widespread use of 2.45 GHz radiation, in particular, is continuing to grow [3], raising issues about potential health risks, especially to the nervous system [4,5,6].

Heat Shock Proteins (HSPs), also known as chaperones or stress proteins, are highly conserved evolutionary proteins [7] found in almost all organisms. They are upregulated in response to various stressors [8,9,10] for cytoprotection through the repair or degradation of damaged proteins [11]. HSP-70 is the most common stress-induced protein in the HSP families [12] and has key neuroprotective roles [11,13,14]. Radiofrequencies are known to influence HSP-70 expression [15,16,17,18].

Glucocorticoid receptors (GCRs) belong to the nuclear hormone receptor superfamily of transcription factors. They regulate various cellular mechanisms by binding with glucocorticoid (GC) hormones [19,20]. GCR is considered a cell-stress biomarker because its expression level can be influenced by many factors, including gender, age [21], or stress [22]. Along these lines, multiple GCR alterations have been observed in rat thymus after exposure to 2.45 GHz signals [23]. GCR is expressed in almost all body organs, including the brain [21,24]. To the best of our knowledge, the effects of such nonionizing radiation on brain GCR have not yet been studied.

Astrocytes are also involved in several cerebral processes [25] and become reactive through overexpression of glial fibrillary acidic protein (GFAP) [26] in cases of stress or injury. GFAP is an intermediate filament protein [27] that is highly specific to astroglial cells [28,29,30,31,32]. Enhanced GFAP expression levels constitute a reliable marker of brain injury [33] and radiofrequencies have been reported to increase GFAP levels [34,35,36].

Several factors, including time, can cause the effects of nonionizing radiation in the brain to vary. Some studies have found in vitro and in vivo expression of HSP-70 to be dependent on the duration of irradiation [37,38]. In another study, nonionizing radiation-induced glia reactivity disappeared 6 and 10 days after exposure [39]. Sensitivity to radiofrequency radiation also seems to vary in different brain regions. In our previous research, the limbic areas showed high vulnerability to nonionizing radiation, causing neuronal activity [40], astrocyte proliferation [41], and a decrease of chaperone HSP-90 [42]. Because these areas contain glucocorticoid receptors [43], we wondered if really the nonionizing radiation could be a physical stress stimulus and the relationship it establishes with the glial population.

In the research presented here, we studied the evolution of HSP-70 and GCR cell-stress biomarkers along with glial activation (GFAP) in rat brain in response to single and repeated exposure to nonthermal 2.45 GHz radiation at different postexposure times: 90 min and 24 h after acute irradiation and 90 min after repeated irradiation. These markers were analyzed using chemiluminescent enzyme-linked immunosorbent assay and immunohistochemistry testing for four key brain regions: the somatosensory cortex, the limbic cortex, the hypothalamus and the hippocampus.

## 2. Results

### 2.1. Heat Shock Protein (HSP) 70 Expression

#### 2.1.1. HSP-70 in the Somatosensory Cortex

Effects in irradiated and control animals were not dependent on exposure frequency or time after exposure. There was no statistically significant (*p* = 0.436) interaction between differences in power levels and the effects of number of exposures and postexposure time.

Nonirradiated, single-exposure rats exhibited significantly (*p* = 0.0001) higher HSP-70 expression in the somatosensory cortex at 90 min postexposure than their control animals 24 h after exposure and repeated-exposure animals. There was no statistically significant difference (*p* = 0.319) between single-exposure 24 h postexposure control animals and repeated-exposure groups.

Irradiated rats exposed to a single dose expressed significantly (*p* = 0.0001) more HSP-70 in the somatosensory cortex at 90 min postexposure than animals tested 24 h after exposure to a single dose and repeated-exposure animals. There was no statistically significant difference (*p* = 0.850) between rats at 24 h postexposure to a single dose and repeated-exposure groups.

**Figure 1:**
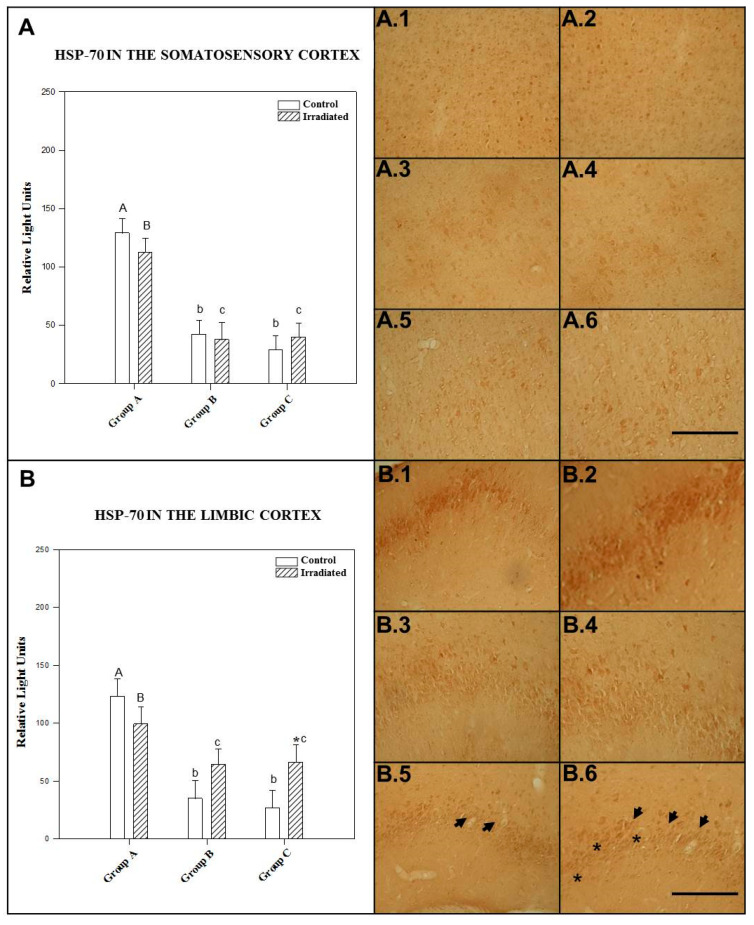
The histograms represent HSP-70 levels detected by ELISA in the somatosensory cortex (**A**) and the limbic cortex (**B**), 1.5 h (Group A) and 24 h (Group B) after acute exposure and 1.5 h after final repeated exposure (Group C) to a 2.45 GHz signal at 0 and 3 W. Each bar represents mean ± SEM (*n* = 24 samples/per group). Asterisks (*) indicate statistically significant differences (*p* < 0.05) between the irradiated and control for each group. a, b, c indicate statistically significant differences (*p* < 0.05) between irradiated or nonirradiated animals comparing between the different groups A, B, C, which were determined using two-way ANOVA followed by the Holm-Sidak test for multiple comparisons The photographs show the HSP-70 immunomarkers for groups A, B and C in the somatosensory cortex, control (A.1, A.3 and A.5) and irradiated animals (A.2, A.4 and A.6); in the limbic cortex, control (B.1, B.3, B.5) and irradiated animals (B.2, B.4, B.6) (*n* = 15 samples/per group). In the photographs it is indicate with the arrowhead and asterisk that means increase on regional cytolocalization. Scale bar = 60 µm.

No significant differences were found among control animals, those tested at 90 min postexposure to a single dose of irradiation (*p* = 0.548), 24 h (*p* = 0.988) and those given repeated exposure (*p* = 0.236) (see Figure 1A).

The mean ± SEM cellular immunoreactivity values for HSP-70 in the somatosensory cortex of the different groups and the significant differences among them can be seen in Table 1 and in the morphology presented in photos A.1, A.2, A.3, A.4, A.5 and A.6 of Figure 1.

#### 2.1.2. HSP-70 in the Limbic Cortex

The effect in irradiated and control rats was not dependent on exposure frequency and postexposure period. There was no statistically significant interaction (*p* = 0.060).

Nonirradiated animals displayed a statistically significant (*p* = 0.0001) increase in HSP-70 expression in the limbic cortex at 90 min postexposure compared to control animals exposed to a single dose of 2.45 GHz irradiation exposure at 24 h postexposure and those that underwent repeated exposure. However, no significant difference (*p* = 0.074) was noted between control rats for single-exposure at 24 h and repeated-exposure groups.

Rats kept alive 90 min after single exposure to 2.45 GHz radiofrequencies showed a statistically significant increase in HSP-70 expression in the limbic cortex compared to single-exposure rats at 24 h postexposure (*p* = 0.022) and repeated-exposure rats (*p* = 0.005).

There was no significant difference (*p* = 0.462) between rats kept alive 24 h after acute irradiation and those exposed to repeated irradiation.

In the limbic cortex, animals exposed repeatedly had significantly (*p* = 0.015) higher HSP-70 expression than their corresponding controls. No statistically significant difference in HSP-70 expression was found after 90 min (*p* = 0.389) or 24 h (*p* = 0.149) between irradiated and control rats for single exposure to 2.45 GHz irradiation (see Figure 1B).

The mean ± SEM cellular immunoreactivity values for HSP-70 in the limbic cortex of the different groups and the significant differences among them can be seen in Table 1 and in the morphology presented in photos B.1, B.2, B.3, B.4, B.5 and B.6 of Figure 1.

#### 2.1.3. HSP-70 in the Hypothalamus

The effect of radiation/no radiation was not dependent on 2.45 GHz radiation exposure frequency or time after irradiation. There was no statistically significant interaction between these factors (*p* = 0.798).

HSP-70 expression in the hypothalamus level was significantly higher in the control group for acute exposure to 2.45 GHz radiation at 90 min postexposure than in control groups for 24 h postexposure (*p* = 0.001) and repeated irradiation (*p* = 0.0001). There was no statistically significant (*p* = 0.304) difference between nonirradiated animals 24 h after single irradiation and those exposed repeatedly to 2.45 GHz radiation.

Acute exposure to 2.45 GHz radiation with 90 min postexposure time induced a statistically significant (*p* = 0.0001) increase of HSP-70 in the hypothalamus compared to groups exposed to single irradiation at 24 h postexposure and those exposed repeatedly to 2.45 GHz radiation. There was no significant (*p* = 0.966) difference between irradiated rats after repeated exposure and those exposed to a single dose at 24 h postexposure.

**Figure 2:**
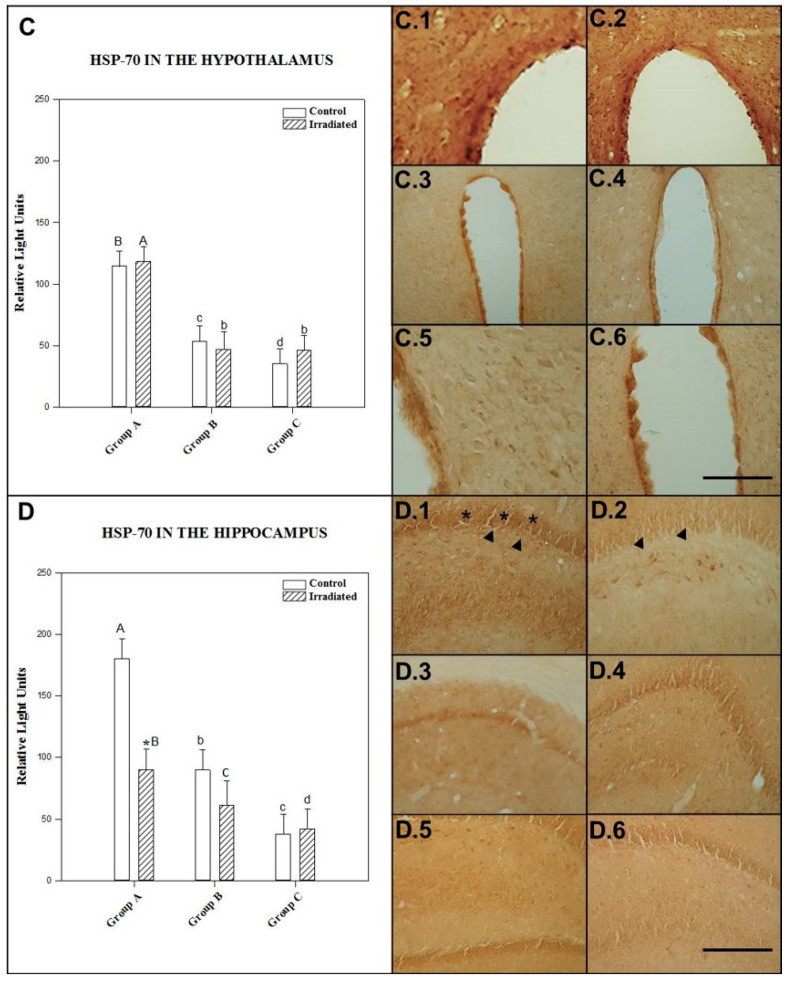
The histograms represent HSP-70 levels detected by ELISA in the hypothalamus (**C**) and the hippocampus (**D**) 1.5 h (Group A) and 24 h (Group B) after acute exposure and 1.5 h after final repeated exposure (Group C) to a 2.45 GHz signal at 0 and 3 W. Each bar represents mean ± SEM (*n* = 24 samples/per group). Asterisks (*) indicate statistically significant differences (*p* < 0.05) between the irradiated and control for each group. a, b, c, d indicate statistically significant differences (*p* < 0.05) between irradiated or nonirradiated animals comparing between the different groups A, B, C, which were determined using two-way ANOVA followed by the Holm-Sidak test for multiple comparisons. The photographs show the HSP-70 immunomarkers for groups A, B and C in the hypothalamus, control (C.1, C.3 and C.5) and irradiated animals (C.2, C.4 and C.6); in the hippocampus, control (D.1, D.3 and D.5) and irradiated animals (D.2, D.4 and D.6) (*n* = 15 samples/per group). In the photos it is indicate with the arrowhead and asterisk that means increase on regional cytolocalization. Scale bar = 60 µm.

No statistically significant difference was observed between irradiated and control rats for single exposure to 2.45 GHz signal at 90 min (*p* = 0.835), at 24 h (*p* = 0.735) or for repeated exposure (*p* = 0.532) (see Figure 2C).

The mean ± SEM cellular immunoreactivity values for HSP-70 in the hypothalamus of the different groups and the significant differences between them can be seen in Table 1 and in the morphology presented in photos C.1, C.2, C.3, C.4, C.5 and C.6 of Figure 2.

#### 2.1.4. HSP-70 in the Hippocampus

The effect of different power levels (0 and 3 W) was not dependent on irradiation frequency or postexposure time. Indeed, there was no statistically significant interaction between the two (*p* = 0.079).

HSP-70 expression in the hippocampus level was significantly higher in control rats for single exposure after 90 min than in nonirradiated animals 24 h after exposure (*p* = 0.006) and controls for the repeated-exposure group (*p* = 0.0001).

In addition, nonirradiated animals given acute exposure with a 24 h postexposure period expressed significantly (*p* = 0.001) more HSP-70 than those of the repeated-exposure group.

Animals kept alive 90 min after single exposure to 2.45 GHz irradiation displayed a statistically significant (*p* = 0.002) increase in HSP-70 expression in the hippocampus compared to the repeatedly exposed group. No significant difference was found between acute-exposure rats at 24 h postexposure and those tested 90 min after acute (*p* = 0.062) or repeated (*p* = 0.331) exposure to 2.45 GHz irradiation.

The rats sacrificed 90 min after acute irradiation expressed significantly (*p* = 0.018) lower HSP-70 in the hippocampus compared to their controls. There was no significant difference between irradiated and control rats exposed to a single dose of 2.45 GHz radiation at 24 h postirradiation (*p* = 0.139) and those subjected to repeated exposure (*p* = 0.487) (see Figure 2D).

The mean ± SEM cellular immunoreactivity values for HSP-70 in the hippocampus of the different groups and the significant differences among them can be seen in Table 1 and in the morphology presented in photos D.1, D.2, D.3, D.4, D.5 and D.6 of Figure 2.

### 2.2. Glucocorticoid Receptor (GCR) Expression

#### 2.2.1. GCR in the Somatosensory Cortex

The effect of irradiation/no irradiation was not dependent on irradiation frequency or postexposure time, since no statistically significant interaction was found between the two factors (*p* = 0.145)

Nonirradiated control animals for single exposure with 90 min postexposure showed significantly higher (*p* = 0.011) GCR expression than control animals in the repeated irradiation group. There was a statistically significant (*p* = 0.033) difference in GCR expression in somatosensory cortex level between control rats tested 90 min after single exposure and those tested 24 h after single exposure.

No significant difference (*p* = 0.527) was observed between control animals for 24 h after acute exposure and those for repeated irradiation.

No statistically significant (*p* > 0.05) difference was found among any groups of irradiated rats (repeat-exposure, single exposure at 90 min (*p* = 0.387) and at 24 h (*p* = 0.143). More specifically, no significant differences were observed between animals sacrificed 90 min and 24 h after single exposure to 2.45 GHz radiation (*p* = 0.485).

**Figure 3:**
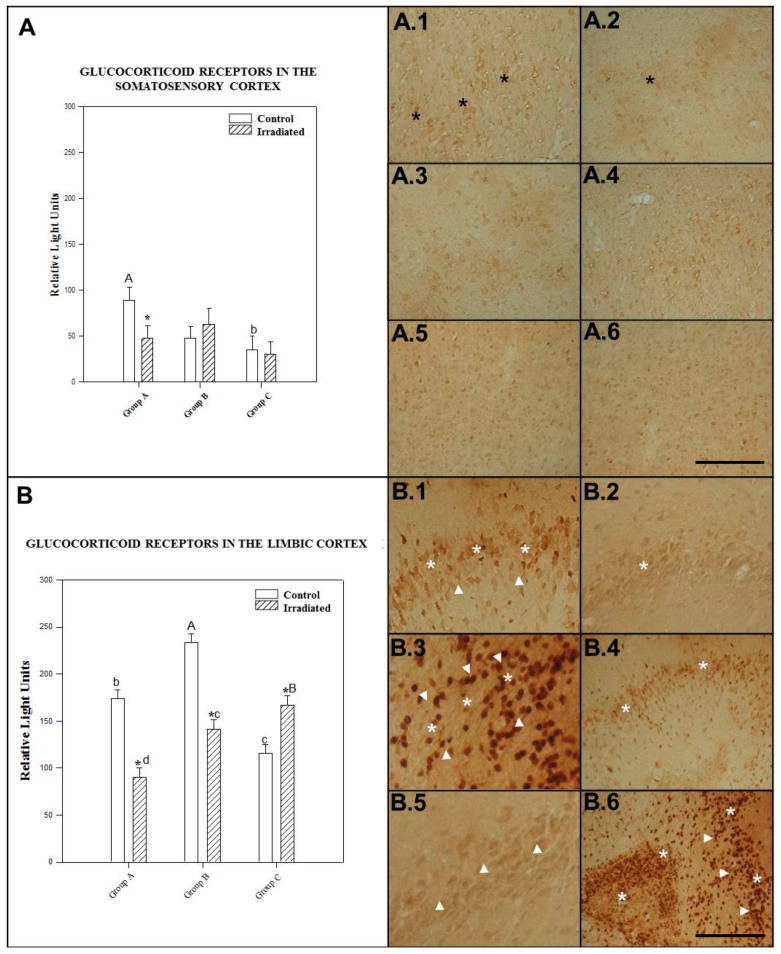
The histograms represent glucocorticoid receptor (GCR) levels detected by ELISA in the somatosensory cortex (**A**) and the limbic cortex (**B**), 1.5 h (Group A) and 24 h (Group B) after acute exposure and 1.5 h after repeated exposure (Group C) to a 2.45 GHz signal at 0 and 3 W. Each bar represents mean ± SEM (*n* = 24 samples/per group). Asterisks (*) indicate statistically significant differences (*p* < 0.05) between the irradiated and control for each group. a, b, c, d indicate statistically significant differences (*p* < 0.05) between irradiated or nonirradiated animals comparing between the different groups A, B, C, which were determined using two-way ANOVA followed by the Holm-Sidak test for multiple comparisons. The photographs show the GCR immunomarkers for groups A, B and C in the somatosensory cortex, control (A.1, A.3 and A.5) and irradiated animals (A.2, A.4 and A.6); in the limbic cortex, control (B.1, B.3 and B.5) and irradiated animals (B.2, B.4 and B.6) (*n* = 15 samples/per group). In the photos it is indicate with the arrowhead and asterisk that means increase on regional cytolocalization. Scale bar = 60 µm.

The animals subjected to acute irradiation with a 2.45 GHz signal and kept alive for 90 min postexposure showed a statistically significant (*p* = 0.037) decrease in GCR expression in the somatosensory cortex compared to their corresponding controls. There was no significant difference between irradiated animals and their controls 24 h after single exposure to 2.45 GHz (*p* = 0.489) or after repeated irradiation (*p* = 0.810) (see Figure 3A).

The mean ± SEM cellular immunoreactivity values for GCR in the somatosensory cortex of the different groups and the significant differences among them can be seen in Table 2 and in the comparative morphology presented in photos A.1, A.2, A.3, A.4, A.5 and A.6 of Figure 3.

#### 2.2.2. GCR in the Limbic Cortex

The effect of different levels of irradiation/no irradiation was dependent on the level of postexposure time. There was a statistically significant interaction between irradiated/nonirradiated animals and postexposure time (*p* ≤ 0.001).

Nonirradiated animals at 24 h after exposure showed significantly higher (*p* = 0.0001) GCR expression than control animals in the repeated-exposure group or those tested 90 min after exposure (*p* = 0.025). A statistically significant (*p* = 0.050) difference in GCR expression in the limbic cortex was found between control rats tested 90 min after single exposure and control rats for repeated exposure.

A statistically significant difference in GCR expression in the limbic cortex was observed between repeatedly irradiated rats and those tested 90 min after exposure to acute irradiation with a 2.45 GHz signal (*p* = 0.0001). A significant difference (*p* = 0.0001) in GCR expression was also found between rats sacrificed 24 h and 90 m after acute irradiation with a 2.45 GHz signal.

There was no significant difference between repeated-exposure rats and those tested 24 h after acute exposure to 2.45 GHz radiation (*p* = 0.073).

Repeated exposure to 2.45 GHz radiation induced a statistically significant (*p* = 0.0001) increase in GCR compared to control rats. There was a significant difference between nonirradiated and irradiated animals tested 90 min or 24 h after single exposure to a 2.45 GHz signal (*p* = 0.0001) (see Figure 3B).

The mean ± SEM cellular immunoreactivity values for GCR in the limbic cortex of the different groups and the significant differences among them can be seen in Table 2 and in the morphology presented in photos B.1, B.2, B.3, B.4, B.5 and B.6 of Figure 3.

#### 2.2.3. GCR in the Hypothalamus

The effect of irradiation/no irradiation was not dependent the number of exposures or postexposure time, since no statistically significant interaction was found between the two factors (*p* = 0.216).

Among control animals, GCR expression in the hypothalamus was significantly higher in those tested 90 min after exposure than in those tested after 24 h (*p* = 0.0001) or repeated irradiation (*p* = 0.005). GCR expression was significantly higher in nonirradiated control animals for repeated exposure (*p* = 0.038) than in those tested 24 h after acute exposure.

No statistically significant differences in GCR expression in the hypothalamus were observed among repeatedly exposed rats and those tested 90 min (*p* = 0.073) or 24 h (*p* = 0.619) after single exposure to irradiation with a 2.45 GHz signal. However, there was a significant difference (*p* = 0.038) in GCR expression between rats sacrificed 90 min and 24 h after single exposure to a 2.45 GHz signal.

GCR expression in the hypothalamus diminished significantly (*p* = 0.005) in animals tested 90 min after exposure to a single dose of irradiation from a 2.45 GHz signal, compared to control animals.

**Figure 4:**
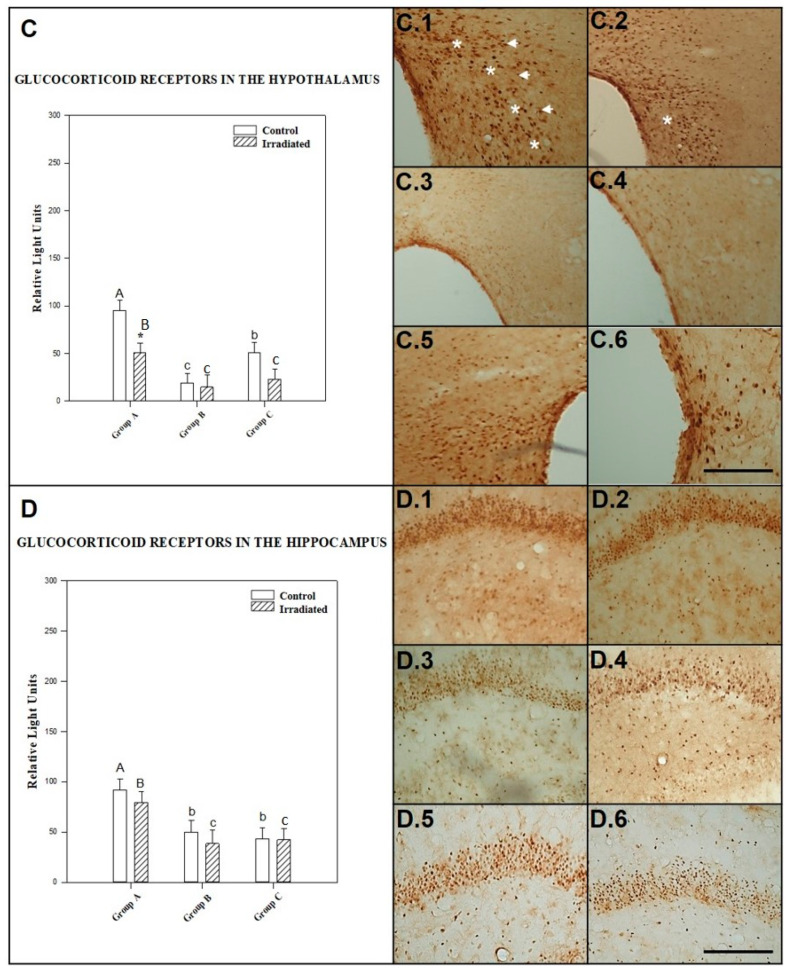
The histograms represent glucocorticoid receptor (GCR) levels detected by ELISA in the hypothalamus (**C**) and the hippocampus (**D**) at 1.5 h (Group A) and 24 h (Group B) after acute exposure and 1.5 h after final repeated exposure (Group C) to a 2.45 GHz signal at 0 and 3 W. Each bar represents mean ± SEM (*n* = 24 samples/per group). Asterisks (*) indicate statistically significant differences (*p* < 0.05) between the irradiated and control for each group. a, b, c indicate statistically significant differences (*p* < 0.05) between irradiated or nonirradiated animals comparing between the different groups A, B, C, which were determined using two-way ANOVA followed by the Holm-Sidak test for multiple comparisons. The photographs show the GCR immunomarkers for group A, B and C in the hypothalamus, control (C.1, C.3 and C.5) and irradiated animals (C.2, C.4 and C.6) in the hippocampus, control (D.1, D.3 and D.5) and irradiated animals (D.2, D.4 and D.6) (*n* = 15 samples/per group). In the photos it is indicate with the arrowhead and asterisk that means increase on regional cytolocalization. Scale bar = 60 µm.

There was no significant difference between irradiated and control groups tested 24 h after acute exposure to 2.45 GHz radiation (*p* = 0.799) and after repeated irradiation (*p* = 0.070) (see Figure 4C).

The mean ± SEM cellular immunoreactivity values for GCR in the hypothalamus of the different groups and the significant differences among them can be seen in Table 2 and in the morphology presented in photos C.1, C.2, C.3, C.4, C.5 and C.6 of Figure 4.

#### 2.2.4. GCR in the Hippocampus

The effect of power levels (0 and 3 W) was not dependent on acute/repeated exposure or postexposure period. Statistically, there was no significant interaction between these factors (*p* = 0.713).

Control rats for acute exposure to 2.45 GHz irradiation and 90 min postexposure time showed significantly higher GCR expression in the hippocampus than nonirradiated control animals for single and 24 h after exposure (*p* = 0.001) and those for repeated exposure (*p* = 0.024). No significant (*p* = 0.258) difference was observed between control animals tested after repeated irradiation and those tested 24 h after acute exposure.

Rats exposed to a single dose of irradiation showed significantly (*p* = 0.003) higher expression of GCR in the hippocampus at 90 min than at 24 h after irradiation. No significant difference was detected between irradiated rats exposed repeatedly to 2.45 GHz radiation and those exposed to a single dose and tested after 90 min (*p* = 0.208) or 24 h (*p* = 0.054).

There was no significant difference between irradiated and control rats among those exposed to a single dose of 2.45 GHz radiation and tested at 90 min (*p* = 0.366) or 24 h (*p* = 0.424) after exposure or after repeated irradiation (*p* = 0.903) (see Figure 4D).

The mean ± SEM cellular immunoreactivity values for GCR in the hippocampus of the different groups and the significant differences among them can be seen in Table 2 and in the morphology presented in photos D.1, D.2, D.3, D.4, D.5 and D.6 of Figure 4.

### 2.3. Glial Fibrillary Acidic Protein (GFAP) Expression

#### 2.3.1. GFAP in the Somatosensory Cortex

The effect of irradiation/no irradiation was not dependent on the number of exposures or time after exposure, since no significant interaction was found between these factors (*p* = 0.361).

GFAP expression in the somatosensory cortex was significantly higher in control rats for 24 h after single exposure than in control rats for 90 min after single exposure (*p* = 0.01) and repeated irradiation (*p* = 0.025). There was no significant (*p* = 0.887) difference in GFAP expression between nonirradiated animals after repeated exposure to a 2.45 GHz signal and those exposed to a single dose and tested after 90 min.

There was no significant difference in GFAP expression in the somatosensory cortex of repeatedly exposed rats compared to single-exposure groups that were kept alive for 90 min (*p* = 0.133) and 24 h (*p* = 0.315) prior to sacrifice. However, a significant difference (*p* = 0.021) was detected between groups tested 90 min and 24 h after acute irradiation.

**Figure 5:**
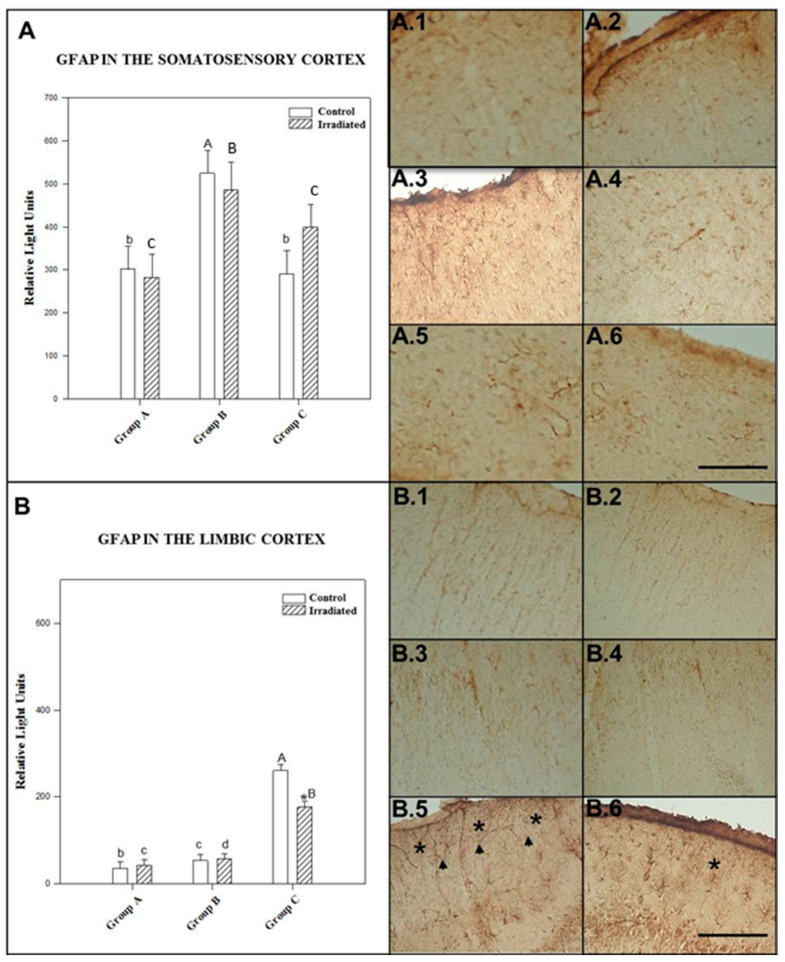
Glial fibrillary acidic protein (GFAP) levels detected by ELISA in the somatosensory cortex (**A**), the limbic cortex (**B**), 1.5 h (Group A) and 24 h (Group B) after acute exposure and 1.5 h after final repeated exposure (Group C) to a 2.45 GHz signal at 0 and 3 W. Each bar represents mean ± SEM. (*n* = 24 samples/per group) Asterisks (*) indicate statistically significant differences (*p* < 0.05) between the irradiated and control for each group. a, b, c, d indicate statistically significant differences (*p* < 0.05) between irradiated or nonirradiated animals comparing between the different groups A, B, C, which were determined using two-way ANOVA followed by the Holm-Sidak test for multiple comparisons. The photographs show the GFAP immunomarkers for groups A, B and C in the somatosensory cortex, control (A.1, A.3 and A.5) and irradiated animals (A.2, A.4 and A.6); in the limbic cortex, control (B.1, B.3 and B.5) and irradiated animals (B.2, B.4 and B.6) (*n* = 15 samples/per group). In the photos it is indicate with the arrowhead and asterisk that means increase on regional cytolocalization. Scale bar = 60 µm.

There was no significant difference (*p* > 0.05) (*p* = 0.804; *p* = 0.652 and *p* = 0.162) in GFAP expression in the somatosensory cortex between irradiated and control rats at any level of exposure to 2.45 GHz irradiation (90 min and 24 h after single exposure and after repeated exposure, respectively) (see Figure 5A).

The mean ± SEM cellular immunoreactivity values for GFAP in the somatosensory cortex of the different groups and the significant differences among them can be seen in Table 3 and in the morphology presented in photos A.1, A.2, A.3, A.4, A.5 and A.6 of Figure 5.

#### 2.3.2. GFAP in the Limbic Cortex

The effect of irradiation/no irradiation was dependent on the number of irradiation sessions and postexposure time. There was a statistically significant (*p* = 0.045) interaction between these factors.

Nonirradiated rats of repeated exposure group showed significantly (*p* = 0.0001) higher GFAP expression in their limbic cortex than control animals of acute exposure (followed by 90 min and 24 h) groups.

Repeatedly exposed rats to 2.45 GHz signal displayed statistically significant (*p* = 0.0001) increase in GFAP expression than acutely irradiated rats with both 90 min and 24 h postexposure period. No significant (*p* = 0.055) difference was noticed between the rats subjected to acute irradiation with 2.45 GHz signal and maintained alive 90 min and 24 h later.

There was no statistically significant difference in GFAP expression at limbic cortex level between irradiated and control animals of single exposure to 2.45 GHz irradiation followed by 90 min (*p* = 0.363) and 24 h (*p* = 0.535) postexposure time. GFAP expression in limbic cortex was significantly (*p* = 0.023) diminished due to repeated exposure to 2.45 GHz signal with regard to control group (see Figure 5B).

The mean ± SEM cellular immunoreactivity values for GFAP in the limbic cortex of the different groups and the significant differences among them can be seen in Table 3 and in the morphology presented in photos B.1, B.2, B.3, B.4, B.5 and B.6 of Figure 5.

#### 2.3.3. GFAP in the Hypothalamus

The effect of irradiation/no irradiation was dependent on the number of irradiation sessions and postexposure time, as there was a statistically significant interaction (*p* = 0.028).

Control rats in the repeated-exposure group showed a statistically significant (*p* = 0.0001) increase in GFAP expression in the hypothalamus compared to nonirradiated animals 90 min and 24 h after single-exposure. Repeatedly irradiated rats expressed significantly (*p* = 0.0001) more GFAP in the hypothalamus than rats exposed once to a 2.45 GHz signal (and tested at 90 min and 24 h postexposure). Increase in GFAP expression was statistically significant (*p* = 0.002) in rats tested 24 h after single exposure compared to those tested 90 min after single exposure to 2.45 GHz irradiation.

**Figure 6:**
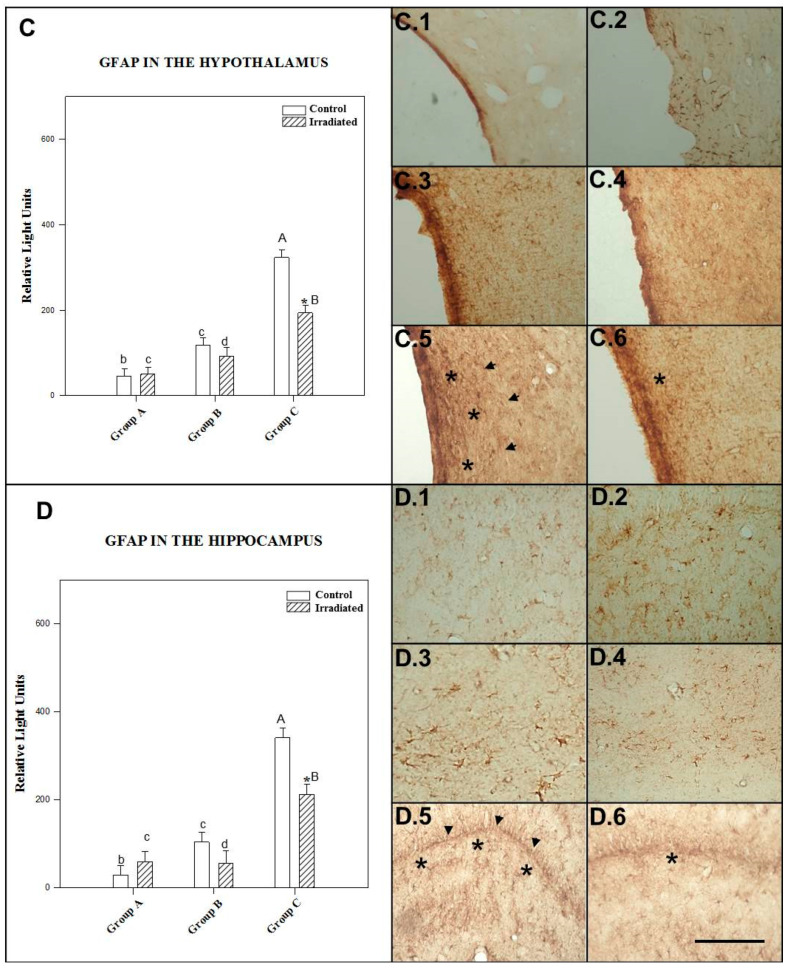
Glial fibrillary acidic protein (GFAP) levels detected by ELISA in the hypothalamus (**C**) and the hippocampus (**D**) 1.5 h (Group A) and 24 h (Group B) after acute exposure and 1.5 h after final repeated exposure (Group C) to a 2.45 GHz signal at 0 and 3 W. Each bar represents mean ± SEM (*n* = 24 samples/per group). Asterisks (*) indicate statistically significant differences (*p* < 0.05) between the irradiated and control for each group. a, b, c, d indicate statistically significant differences (*p* < 0.05) between irradiated or nonirradiated animals comparing between the different groups A, B, C, which were determined using two-way ANOVA followed by the Holm-Sidak test for multiple comparisons. The photographs show the GFAP immunomarkers for groups A, B and C in hypothalamus, control (C.1, C.3 and C.5) and irradiated animals (C.2, C.4 and C.6); in hippocampus, control (D.1, D.3 and D.5) and irradiated animals (D.2, D.4 and D.6) (*n* = 15 samples/per group). In the photos it is indicate with the arrowhead and asterisk that means increase on regional cytolocalization. Scale bar = 60 µm.

Only repeated exposure to 2.45 GHz irradiation triggered a statistically significant decrease in GFAP expression compared to the nonirradiated group (*p* = 0.006). There was no significant difference between irradiated and control animals tested at 90 min (*p* = 0.302) and 24 h (*p* = 0.216) after acute exposure (see Figure 6C).

The mean ± SEM cellular immunoreactivity values for GFAP in the hypothalamus of the different groups and the significant differences among them can be seen in Table 3 and in the morphology presented in photos C.1, C.2, C.3, C.4, C.5 and C.6 of Figure 6.

#### 2.3.4. GFAP in the Hippocampus

The effect of power levels (0 and 3 W) was dependent on the number of irradiation sessions and postexposure time, as there was a statistically significant interaction (*p* = 0.005).

Control animals for repeated exposure to 2.45 GHz irradiation showed a statistically significant (*p* = 0.0001) increase in GFAP expression in the hippocampus compared to nonirradiated animals tested 90 min and 24 h after single exposure. GFAP expression was significantly (*p* = 0.025) higher in the hippocampus of control rats tested 24 h after single exposure than in those tested 90 min after single exposure.

Repeatedly irradiated rats presented a significant (*p* = 0.0001) increase in GFAP expression in the hippocampus compared to rats kept alive for 90 min and 24 h after acute irradiation. No significant (*p* = 0.928) difference was found between irradiated animals tested 90 min and 24 h after single exposure to irradiation.

Only rats subjected to repeated exposure to 2.45 GHz irradiation showed a significant (*p* = 0.0001) decrease in GFAP expression in the hippocampus compared to the nonirradiated group. No significant difference was found between irradiated and control rats tested 90 min (*p* = 0.343) and 24 h (*p* = 0.197) after acute exposure (see Figure 6D).

The mean ± SEM cellular immunoreactivity values for GFAP in the hippocampus of the different groups and the significant differences among them can be seen in Table 3 and in the morphology presented in photos D.1, D.2, D.3, D.4, D.5 and D.6 of Figure 6.

## 3. Discussion

We observed that the stress biomarkers HSP-70 and GCR exhibited parallel behavior in most of the regions studied and presented lower levels than the GFAP astrocyte activation biomarker after irradiation, but were not significantly different or even lower than those of nonradiated animals in all the regions studied.

### 3.1. Influence and Temporal Evolution of 2.45 GHz Radiation on HSP-70 and GCR in Brain Tissues

In this experimental study, we found signs of stress from repeated irradiation in the limbic cortex, along with hypo-suppression and/or unresponsiveness due to decreases in GCR and the chaperone that assists the intracellular trafficking of GCR/HSP-70 in all other brain regions.

Changes in the pattern and intensity of GCR and HSP-70 after single or repeated irradiation in the anatomical brain regions studied resembled what Filipovic [44] described for acute and chronic stress in areas of the cerebrum.

High levels of this protein are reported for animals which have been suffering immobilization (controls) after 90 min, descending after 24 h. This time after the stimulus, as pointed out by some authors [45,46], can be relevant even if it has a different nature (radiation or immobilization). In the same manner, the accustomization to the stress provoked by the repeated immobilization or radiation can be clearly emphasized, motivated on an important descend of the HSP-70 on each one of the studied areas [47]. These results are in line with findings from our previous research on the thyroid gland, in which peak expression of HSP-70 occurred 90 min after irradiation and recovery was generally established after 24 h [48]. In another study, acute exposure to a 2.45 GHz signal at subthermal SAR levels and at different powers triggered an imbalance in cerebral anatomical HSP-90 (alpha/beta) that was compensated by the antiapoptotic mechanism [42]. Findings from other authors suggest that modulation of heat shock protein expression in neuronal cells could be an early response to radiofrequency microwaves [49].

Repeated exposure to 2.45 GHz irradiation significantly (*p* = 0.015) elevated HSP-70 levels in the limbic cortex of irradiated rats compared to nonirradiated rats. This result indicates that although HSP-70 production in response to acute irradiation stress is initially similar to the stress caused by immobilization in most rat brain areas, repeated exposure to radiofrequencies limbic cortex caused an increase in cytoprotection compared to nonirradiated animals. The vulnerability of the limbic cortex to nonionizing radiation has been described in prior research [40]. The effects of the stress are more visible in this anatomical area due to its GCR content [43]. Thus, increased numbers of GCR in the limbic cortex may indicate future dysfunction, because GCR malfunction is involved in the pathogenesis of various diseases, including major depressive disorder [50] and cognitive deficit [51].

In other research recovery of HSP-90 the limbic cortex occurred later than in the other brain regions, at 24 h after irradiation at 2.45 GHz in rats [42]. However, repeated exposure to mobile phone irradiation led to continuous expression of HSPs in exposed cells and tissues, which affects their normal regulation and promotes cancer [52].

Interestingly, acute 2.45 GHz signal exposure induced a significant decrease (*p* = 0.018) in HSP-70 expression in the hippocampus compared to nonirradiated animals. This effect was also observed following acute stress [46,53], and 24 h after acute irradiation [42], in relation to GCR in the brain of acutely stressed rats [44]. It could be due to the insufficient duration of the radiofrequency stimulation to increase HSP-70 levels [37,38,54] and the greater sensitivity of the hippocampus to other stress stimuli such as acute immobilization [55].

Our finding and those of other authors indicate that HSP-70 expression increased for cytoprotection in most cerebral areas in rats tested 90 min after exposure to acute stress from irradiation or immobilization for 1 h or 2 h [18,56].

Repeated exposure to irradiation; however, decreased HSP-70 expression compared to single exposure. In related research, coordinated interactions between HSP-90 and HSP-70 also enhanced GCR stability, function and regulation [57] while chronic, unpredictable stress dissociated the GCR-HSP complex in rat hippocampus [44].

Interestingly, we found that exposure to acute 2.45 GHz irradiation caused GCR levels to decrease significantly in in both the somatosensory cortex and the hypothalamus of irradiated rats with respect to immobilized ones. Neuroendocrine neurons respond to physiological perturbations by releasing corticotropin-releasing factor (CRF) [58]. Furthermore, vasopressin (AVP) is required for the HPA response to acute stimuli [59]. Both CRF and AVP are essential for the coordination of behavioral and metabolic responses to stress [50]. Some authors reported an influence of millimeter-wave electromagnetic emission on vasopressin expression that was dependent on exposure to irradiation, which suggests that vasopressin may act in the initial response to acute irradiation stimulation [60]. However, the stress response in the female rats used in that experiment was described by other authors to be different than in males [18,56]. This leads us to think that negative feedback may influence the somatosensory cortex and hypothalamus via GCR in the acute response of female rats to stress. This is evident in their increased corticoids and ACTH levels compared to males, after exposure to multiple stressors with different modalities [61].

Hence, we can conclude that repeated exposures to 2.45 GHz radiation could favor pathologies or decompensate physiological conditions [51,62,63,64,65].

### 3.2. The Influence and Temporal Evolution of 2.45 GHz Radiation on GFAP with the Decrease of GCR/HSP-70

As for glia response, repeated exposure to 2.45 GHz signal induced a significant increase in GFAP expression in the limbic cortex, the hypothalamus and the hippocampus compared to single exposure at 90 min and 24 h, but not compared to controls.

Chronic exposure (for 1 and 3 months) to 835 MHz radiofrequencies increased GFAP immunoreactivity in the hippocampus and changed the morphology of the astrocytes in mice at SARs of 1.6 and 4 W/Kg, with a maximal effect at 4 W/Kg [66]. Such an increase in irradiation-induced astrocyte reactivity may have various detrimental consequences, including brain aging [67,68] and neural damage (cell death, synapse loss, axon and myelin damage) [35].

In contrast, other researchers reported no effects from radiofrequencies on GFAP expression after acute [69] or chronic [70] exposure at different rat ages and SARs. Astrocyte reactivation (GFAP upregulation) depended on age, gonadal hormones [69,71] and glucocorticoid levels [72].

All classes of glia cells, including astrocytes, express GCR [73]. As a critical stress-responding transcriptional factor in astrocytes, GCR may be crucially involved in astrocyte activity and may mediate stress-induced adaptation [74].

There is substantial evidence to indicate that glucocorticoid response and glia response are negatively correlated [75]. Indeed, adrenalectomy was found to induce apoptosis and increase glial response by augmenting GFAP expression in adult rats [68], while glucocorticoids (corticosterone treatment) prevented apoptosis by decreasing glial activation [72]. Moreover, GFAP transcription is under the negative regulation of glucocorticoids, suggesting that GCR might be used for in vivo inhibition of the GFAP gene to investigate the role of GFAP in central nervous system damage [76].

We found increased astrocyte proliferation in the limbic cortex, the hypothalamus and the hippocampus of repeatedly irradiated rats compared to acutely exposed rats (see Figure 5). Like many signaling proteins, GCR depends on HSP-90 for in vivo function [77].

HSP-70 overproduction protects against multiple apoptotic stimuli [78] and influences irradiation-induced immunomodulation [79]. However, our results showed decreased HSP-70 levels in all brain areas tested except the limbic cortex. This indicates that the autoimmunity generated by the cellular stress response to irradiation varies according to the cell region affected.

Reactive astrocytosis involving the activation, hypertrophy and proliferation of astrocytes is a characteristic response of the central nervous system to inflammation or injury [80]. It plays an important role as a source of inflammatory cytokines, including TNF-α and IL-1β [81]. This is necessary for maintaining the proper balance between inflammatory responses, glial reactivity and brain repair [72].

## 4. Material and Methods

### 4.1. Experimental Radiation System Protocol

The experimental rat (R) was positioned in a region where maximum field uniformity was guaranteed in the GTEM chamber. The rats were immobilized once or repeatedly in a methacrylate holder for 30 min. The experimental system used for radiating the rats is described in [42]. The SAR values were estimated with the aid of SEM-CAD X [82] and an FDTD-based software tool.

The mean power absorbed by the rats in the four groups was estimated by Equations (1)–(3), as well as the average weight, mean SAR ± SEM in brain and body, and peak SAR ± SEM, averaged for 1 g of brain or body and 2.45 GHz frequency in a Schaffner GTEM cell. The field value E was specified by considering

(1) $$E=\sqrt{Z_{0}P/\left(h^{2}\zeta \right)}$$

where, h is the septum height in the exposure zone (position of the MH), $P_{\mathrm{TR}}$ is the input power on the GTEM cell,

(2) $$P_{\mathrm{TR}}=P_{\mathrm{IN}}-P_{\mathrm{REF}}$$

where $Z_{0}=50\;\Omega$ the input impedance of the cell, and ζ is coefficient which depend of the ripple of the field in the position el MH, is set as 2 [42]. The simulations were performed at 2.45 GHz (by use of a desktop PC with an Intel Core 2 Quad processor working at 2.40 GHz, and with 4 GB RAM available); the corresponding grid positions composed of 6,762,000 voxels (minimum grid step of 0.7 mm and maximum grid step of 3 mm), for computation time of 3.5 h. The SARs were estimated by applying a correction factor to the values obtained from the numerical simulations, in proportion to the ratio between the weight of the model rat and the weights of the experimental rats, as specified by the following expression:

(3) $${\mathrm{SAR}}_{E}={\mathrm{SAR}}_{S}\times W_{s}/W_{E}$$

where ${\mathrm{SAR}}_{E}$ is the estimated value of the experimental SAR, ${\mathrm{SAR}}_{S}$ the SAR obtained during the simulation, $W_{S}=198.3\;\left[g\right]$ the weight of the model rat (see above), and $W_{E}\;\left[g\right]$ the weight of the experimental rat.

Table 4 shows that mean and peak SAR values are directly proportional to the input power for each subgroup. All SAR values were low, and all were below thermal value.

### 4.2. Experimental Design

All experiments were carried out in compliance with the Spanish Directive (RD53/2013), the European Communities Council Directives (2010/63/EU and 86/609/CEE) [83], the ARRIVE guidelines and the UK Animals (Scientific Procedures) Act of 1986 and associated guidelines. All experimental protocols were approved by the University of Santiago de Compostela (USC) Committee on Institutional Bioethics of Animal Care and Use (15005AE/11/FUN.01). Every effort was made to minimize the number of animals used in the study and their suffering.

A total of 54 adult female Sprague-Dawley rats were used in this study. They were randomly assigned to 3 groups (*n* = 18), each of which was divided each into 2 subgroups (*n* = 9) as follows:

Group A: Rats in one subgroup, nonirradiated (control group) dose of 0 W, while the other subgroup was exposed to 3.0 W of 2.45 GHz radiation for 30 min. Both groups were euthanized after 1.5 h (90 min) for tissue extraction. An injectable (i.p.) euthanasia agent (sodium pentobarbital) was administered (the euthanasia dose is 3 times the anesthetic dose).

Group B: One subgroup of nonirradiated rats (control group) dose 0 W and other to 3.0 W radiation at a frequency of 2.45 GHz for 30 min. The rats were kept alive for 24 h and then euthanized by means of an injectable (i.p.) euthanasia agent (sodium pentobarbital, the euthanasia dose is 3 times the anesthetic dose) prior to tissue extraction.

Group C: One subgroup of rats was not irradiated (control group) dose 0 W and other to 3 W of 2.45 GHz radiation for 30 min per day and for 10 days in a period of two weeks. Radiating the animals 5 days per week and resting on the weekends of the exposure. On the last day, the rats were kept alive for 1.5 h (90 min) after irradiation. Then, an injectable (i.p.) euthanasia agent (sodium pentobarbital) was administered (the euthanasia dose is 3 times the anesthetic dose) prior to tissue extraction.

**Figure 7:**
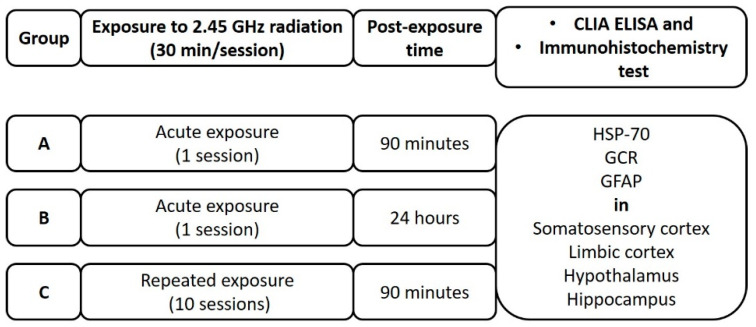
Experimental design. HSP-70: Heat shock protein-70, GCR: glucocorticoid receptor, GFAP: Glial fibrillary acidic protein, CLIA: Chemiluminescent Enzyme-Linked Immunosorbent Assay, Immunohistochemistry.

A diagram of the experimental design is shown in Figure 7.

#### 4.2.1. Tissue Extraction and Preparation of Cell Extracts for the ELISA Technique

After exposure to radiation, the rats were kept alive for the specified amounts of time and then given a lethal dose of Pentothal. Tissue samples from the somatosensory cortex, limbic cortex, hypothalamus and hippocampus were then dissected out under a stereomicroscope (Nikon Eclipse CFI60). The samples were placed in 500 μL of phosphate buffer (PBS; 0.015 M phosphate buffer, 0.15 M NaCl, pH 7.2) containing 0.1 mM pepstatin A, 0.02 mM N-(trans-Epoxysuccinyl)-L-leucine-4 guanidinobutylamide (E-64), 1 mM phenylmethanesulfonyl fluoride (PMSF) and 2 mM ethylenediaminetetraacetic acid (EDTA) protease inhibitors (all from Sigma-Aldrich, Munich, Germany). The samples were disaggregated and homogenized in a Polytron tissue homogenizer (Kinematica AG, Littau, Luzern, Switzerland) at 35,000 r.p.m. for 5 min on ice, and then ultrasonically lysed in a Branson W-250 sonifier (Branson Ultrasonic Corporation, Brookfield, CT, USA) by means of five 10-pulse sonication cycles with a 50% duty cycle output. The whole process was performed on ice. The lysate obtained was centrifuged at 15,000× *g* for 10 min at 4 °C. The supernatant was then aliquoted and frozen at −80 °C until use.

##### Determination of Protein Concentration

Protein concentration in the tissue extracts was determined by the Bradford method and a Bio-Rad Protein Assay kit (BioRad Laboratories, Feldkirchen, Germany), using BSA (Sigma-Aldrich, Munich, Germany) as standard.

##### Chemiluminescent Enzyme-Linked Immunosorbent Assay (CLIA ELISA)

A CLIA test was applied to detect HSP-70, GCR and GFAP. One-microgram (μg) aliquots of protein extract in 100 μL of carbonate-bicarbonate buffer (pH 9.6) were placed in the 96 wells of standard ELISA microplates (Greiner Bio-One High-Binding) and incubated overnight at 4 °C. The plates were then washed three times with TBS (50 mM Tris, 0.15 M NaCl, pH 7.4), blocked for 1 h with TBS containing 0.2% Tween 20 (TBS-T1) and 5% nonfat dry milk, incubated for 2 h at 37 °C with 100 μL of a 1:100 dilution (in TBS-T1 containing 1% nonfat dry milk) of rabbit polyclonal antibodies, antiHSP-70 and glucocorticoid receptor (Santa Cruz Biotechnology, Dallas, Texas, USA) and GFAP (DAKO, Glostrup, Hovedstaden, Denmark), then washed five times with TBS containing 0.05% Tween 20. To detect rabbit immunoglobulins (Ig), 100 μL of polyclonal antibody peroxidase-conjugated goat antirabbit Ig (DAKO) was diluted 1:1000 in TBS-T1and incubated for 1 h at 37 °C. The wells were washed five times in TBS, then treated with 100 μL of enhanced luminol-based chemiluminescent substrate to detect horseradish peroxidase (Pierce ECL Werten Blotting substrate, Thermo Scientific, Waltham, Massachusetts, USA). After incubation for 3 min at 37 °C, plate luminescence was read in a fluorometer/luminometer (FLx800, Biotek, Winooski, VT, USA), and the results were expressed in relative light units (RLU).

#### 4.2.2. Morphological Studies with Immunohistochemistry

##### Perfusion and Tissue Processing

Once the animals were profoundly anesthetized with Pentothal, they were transcardially perfused via the ascending aorta, first with 0.9% saline at 37 °C for 30 s to wash the blood vessels and after with cold 4% paraformaldehyde in 0.1 M phosphate buffer, pH 7.4. The brains were extracted, washed and cryoprotected with a 20–30% of sucrose (in 0.1 M phosphate buffer, pH 7.4′), then sliced into 40-micron sections using a freezing microtome.

##### Immunohistochemistry

First, the sections were preincubated for one hour in a normal serum of the same animal species from which the secondary serum had been obtained (horse), diluted to 10% in 0.02 M potassium phosphate buffered saline (KPBS) with 0.25% or 0.30% Triton X-100. The sections were then incubated all night with the primary antibody: rabbit polyclonal serums HSP-70 (1:500), glucocorticoid antireceptors (1:100) or antiGFAP (1:1000). The next day, the samples were rinsed three times with KPBS, incubated for 30 min with an avidin–biotin–peroxidase complex prepared according to the manufacturer’s instructions (DAKO) or an EnVision Doublestain System Rabbit/Mouse kit (DAKO) and labeled with 3,3-diaminobenzidine (DAB) in imidazole–HCl-buffered H2O2 solution (DAKO). Finally, the sections were mounted on slides, dehydrated with 70°, 96° and 100° alcohol, submerged in Xylol, covered and code-labelled pending microscope examination.

#### 4.2.3. Quantification and Statistical Analysis

Cellular expression (HSP-70, GCR and GFAP) was measured at the 9.2-mm level (interaural coordinates) in cortical areas (somatosensory and limbic), at a transverse section of rat brain, at 7.20 mm interaural coordinates showing the areas within the paraventricular nucleus (PVN) of the hypothalamus, and at the 5.7-mm level in hippocampal areas (dentate gyrus) (Paxinos and Watson, 1986). The researchers who took the positive cell counts were blind to exposure conditions. Three or four brain sections of each of the cortical areas, hypothalamus and hippocampal structures (dentate gyrus) from each rat were examined. To examine localized cellular expression in each area, cells were counted in a magnified 20× using a Nikon Eclipse E200 microscope connected to a computer with morphometric software Counts per field in each of the areas were expressed as the averages of individual animals or experiments EEM per group.

The results shown in the text and in Figure 1, Figure 2, Figure 3, Figure 4, Figure 5 and Figure 6 are expressed as means ± SEM; significant differences (*p* < 0.05).

The CLIA ELISA and Immunohistochemistry results (Figure 1, Figure 2, Figure 3, Figure 4, Figure 5 and Figure 6) to detect HSP-70, GCR and GFAP in four brain regions, as well as polyclonal antibody were determined by two-way ANOVA, based on protein radiation concentration and considering irradiation (no irradiation, irradiation) and time (90 min, 24 h and 10 days) separately for each area (somatosensory cortex, limbic cortex, hypothalamus and hippocampus). The Holm-Sidak test for multiple comparisons was subsequently applied. Natural logarithm transformations were applied to the data as needed to obtain normality and homoscedasticity.

## 5. Conclusions

In conclusion, our results showed that repeated exposure to 2.45 GHz caused dysregulation of GCR/HSP-70, triggering a state of stress that could decrease anti-inflammatory action without favoring glial proliferation. Further research is needed to determine whether the dysfunction generated by repeated irradiation at 2.45 GHz may be a causal factor in the pathogenesis of nervous system disorders.

## Figures and Tables

**Table 1:** Mean values ± SEM of cell counts that were positive for HSP-70 in brain areas: somatosensory cortex (SMS CORTEX), limbic cortex (L. CORTEX), hypothalamus (HIPOTHA), and hippocampus (HIPPCAM), with radiation and without radiation.

		Single Exposure (after 1.5 h)	Single Exposure (after 24 h)	Repeated Exposure
SMS CORTEX	NO RAD	129 ± 16 ^b,c^	42 ± 11 ^a^	29 ± 5 ^a^
	RAD	112 ± 18 ^b,c^	37 ± 9 ^a^	39 ± 6 ^a^
L. CORTEX	NO RAD	123 ± 16 ^b,c^	34 ± 2 ^a^	26 ± 5 ^a^
	RAD	99 ± 18 ^b,c^	64 ± 13 ^a^	66 ± 22 *^,a^
HIPOTHA	NO RAD	114 ± 20 ^b,c^	53 ± 7 ^a,c^	34 ± 11 ^a,b^
	RAD	117 ± 15 ^b,c^	46 ± 14 ^a^	45 ± 5 ^a^
HIPPCAM	NO RAD	179 ± 30 ^b,c^	89 ± 17 ^a,c^	37 ± 7 ^a,b^
	RAD	90 ± 8 *^,c^	60 ± 20	41 ± 6 ^a^

Two-way ANOVA (radiation × time) for each area: somatosensory cortex (SMS CORTEX), limbic cortex (L. CORTEX), hypothalamus (HIPOTHA) and hippocampus (HIPPCAM) (*n* = 15 samples/per group). * indicates significant differences between nonirradiated/irradiated animals within each column in each brain area studied. ^a,b,c^ indicate significant differences between nonirradiated/irradiated animals within each row in each brain area after 1.5 h (a), 24 h (b) or with repeated exposure (c).

**Table 2:** Mean values ± SEM of cell counts that were positive for glucocorticoid receptors (GCR) in the following brain areas: somatosensory cortex (SMS CORTEX), limbic cortex (L. CORTEX), hypothalamus (HIPOTHA) and hippocampus (HIPPCAM), with radiation and without radiation.

		Single Exposure (after 1.5 h)	Single Exposure (after 24 h)	Repeated Exposure
SMS CORTEX	NO RAD	89 ± 24 ^c^	45 ± 10	35 ± 6 ^a^
	RAD	47 ± 14 *^,c^	62 ± 17	30 ± 4 ^a^
L. CORTEX	NO RAD	174 ± 7 ^b,c^	233 ± 7 ^a,c^	115 ± 8 ^a,b^
	RAD	90 ± 7 *^,b,c^	141 ± 9 *^a^	167 ± 9 *^a^
HIPOTHA	NO RAD	95 ± 16 ^b,c^	18 ± 10 ^a,c^	50 ± 11 ^a,b^
	RAD	50 ± 8 *	14 ± 9	23 ± 4
HIPPCAM	NO RAD	91 ± 15 ^b,c^	49 ± 11 ^a^	42 ± 7 ^a^
	RAD	78 ± 14 ^b^	38 ± 13 ^a^	41 ± 4 ^a^

Two-way ANOVA (radiation × time) for each area: somatosensory cortex (SMS CORTEX), limbic cortex (L. CORTEX), hypothalamus (HIPOTHA) and hippocampus (HIPPCAM) (*n* = 15 samples/per group). * indicates significant differences between nonirradiated/irradiated animals within each column in each brain area studied. ^a,b,c^ indicate significant differences between nonirradiated/irradiated animals within each row in each brain area after 1.5 h (a), 24 h (b) or with repeated exposure (c).

**Table 3:** Mean values ± SEM of cell counts that were positive for glial fibrillary acidic protein (GFAP) in brain areas: somatosensory cortex (SMS CORTEX), limbic cortex (L. CORTEX), hypothalamus (HIPOTHA) and hippocampus (HIPPCAM), with radiation and without radiation.

		Single Exposure (after 1.5 h)	Single Exposure (after 24 h)	Repeated Exposure
SMS CORTEX	NO RAD	30 ± 2 ^b^	54 ± 5 ^a^	29 ± 5
	RAD	28 ± 3 ^b^	48 ± 10 ^a^	39 ± 6
L. CORTEX	NO RAD	35 ± 3 ^b,c^	53 ± 7 ^a,c^	260 ± 27 ^a,b^
	RAD	42 ± 5 ^c^	57 ± 5 ^c^	175 ± 20 *^,b^
HIPOTHA	NO RAD	44 ± 6 ^b,c^	117 ± 14 ^a,c^	32 ± 3 ^a,b^
	RAD	49 ± 4 ^b,c^	91 ± 11 ^a,c^	19 ± 10 *^,a,b^
HIPPCAM	NO RAD	27 ± 4 ^b,c^	103 ± 13 ^a,c^	340 ± 50 ^a,b^
	RAD	59 ± 4 *^,c^	55 ± 4 *^,c^	211 ± 15 ^a,b^

Two-way ANOVA (radiation × time) for each area: somatosensory cortex (SMS CORTEX), limbic cortex (L. CORTEX), hypothalamus (HIPOTHA) and hippocampus (HIPPCAM) (*n* = 15 samples/per group). * indicates significant differences between nonirradiated/irradiated animals within each column in each brain area studied. ^a,b,c^ indicate significant differences between nonirradiated/irradiated animals within each row in each brain area after 1.5 h (a), 24 h (b) or with repeated exposure (c).

**Table 4:** Brain and body SAR values for the experimental rats, calculated from the power (P) and electrical field (E).

Experimental Measurement of Specific Absorption Rate by FDTD							
W_E_ [g]	P_TR_ [W]	E [V/m]	Time Elapsed until Perfusion [h]	SAR_E_ IN BRAIN		SAR_E_ IN BODY	
				Mean [W/kg]	Peak 1 g	Mean [W/kg]	Peak 1 g
227.10	3.00	40.28	Single exposure 1.5	0.069420	0.085499	0.040445	0.199947
219.90			Single exposure 24	0.071551	0.088124	0.041687	0.206086
279.20			Repeated exposure 1.5	0.07509	0.09249	0.04375	0.21629

## Data Availability

Data is contained within the article.
